# Treatment of COVID-19 Patients in Italy: A Physician’s Experience and Insights

**DOI:** 10.5041/RMMJ.10419

**Published:** 2020-07-31

**Authors:** Vered Gilad, Giovanni Masoero

**Affiliations:** Cardiothoracic and Vascular Department, San Martino Hospital, IRCCS for Oncology and Neurosciences, Genoa, Italy

**Keywords:** Coronavirus, COVID-19, Italy

## Abstract

The outbreak of coronavirus disease 2019 (COVID-19) in Italy, the first Western country hit by the pandemic, seriously impacted the Italian healthcare system and social and economic environment. This perspective piece focuses on the main challenges faced by Italian hospital managements: hospital overcrowding; the need for urgent reorganization of the country’s healthcare systems; the lack of data regarding COVID-19 diagnostics, clinical course, and effective treatment; individual and collective consequences of the crisis; and the importance of disease containment measures and early treatment strategies.

## INTRODUCTION

February 21, 2020 marked the beginning of the worst healthcare system crisis of the last 100 years in Italy. When the first case of “community-acquired” coronavirus disease 2019 (COVID-19) was diagnosed,^1^ Italy—the first Western country facing the new virus outbreak—was caught unprepared and did not entirely and properly foresee the subsequent events. The lack of understanding of the asymptomatic or pre-symptomatic carrier state of the virus,^2^,^3^ together with an underestimation of its virulence, an exaggerated concern related to public opinion, and the potential for economic damage, led to the loss of precious time and widespread dissemination of the infection.

Written by two physicians working within an Italian hospital, this paper provides an important perspective based on first-hand experience, regarding the challenges faced by Italy’s healthcare system during the COVID-19 crisis.

## HOSPITAL OVERCROWDING AND REORGANIZATION

Shortly after the first patient was diagnosed with COVID-19, Italy witnessed a rapidly increasing number of emergency department referrals; many of the patients were in a critical condition, needing intensive treatment and mechanical ventilation. Recognizing that the number of patients requiring hospitalization was much higher than hospital capacities led to a rapid reorganization of activity. All non-urgent and elective procedures were immediately suspended and routine outpatient clinic activity cancelled, leaving many chronic patients without adequate medical assistance. The number of dedicated COVID-19 hospitalization departments grew rapidly, with some hospitals becoming fully dedicated to treatment of only new pandemic victims. Within a few days, the number of hospital intensive care beds was at least doubled, operating rooms became small intensive care units (ICUs), and emergency departments were transformed into sub-ICUs, enabling non-invasive and mechanical ventilation of increasing numbers of patients. Due to a shortage of healthcare workers (HCW) in many hospitals, specialists from different departments and resident doctors were transferred to COVID-19 departments, and external specialists were hired to help with the growing workload.

Despite the eventual adoption of the above measures, in areas with early spread and high contagion rate of the virus, treatment was frequently inadequate due to extreme overcrowding. Many hospitals had an insufficient number of ICU beds due to the rising demand, forcing doctors to make difficult ethical decisions, and an unknown number of patients remained undertreated.^4^ Many patients were sent home despite displaying evident signs of pneumonia, and many others died before reaching the hospitals. Treatment delay led to advanced pulmonary and multi-organ damage and was often fatal, contributing to the high number of deaths in Italy—33,900 patients as of July 2020, corresponding to about 14% of diagnosed cases.^5^

Virus spread was highly variable among the regions in Italy, resulting in significant divergence in COVID-19 and overall mortality rates. For example, Bergamo, a city where the virus spread widely in the early stage, had a prevalence of 1.3% with a 16.2% COVID-19 mortality rate, and 6,238 total deaths between February 20 and March 31, 2020, corresponding to a mortality rate that increased by 400% compared to the same time period in previous years (2015–2019).^6^,^7^ Conversely, during the same time, Rome, as a result of early lockdown, had a prevalence of 0.1%, and significantly lower mortality rates, with 133 COVID-19 deaths (2.2%) and an overall decrease in mortality (−8%) compared to the same time period in 2015–2019.^6^,^7^

## INDIVIDUAL AND COLLECTIVE CONSEQUENCES

However, the tragedy extended far beyond numbers; owing to local precautionary isolation measures, patients often faced the disease completely alone. Contact between cities and regions was limited, and many families remained separated for many weeks. Throughout Italy, during lockdown relatives were not allowed to enter the hospitals, and information about clinical status and communication with patients were possible only via telephone and electronic technology. Families lost their loved ones without any possibility to say farewell, nor to even hold funerals, which were suspended until the beginning of May (and for which attendance was then strictly limited to only a small number of relatives, no more than 15 people).

The incidence of anxiety and stress disorders was high among HCW; a recent cross-sectional study reported a high prevalence of post-traumatic stress symptoms (49.4%), depression (24.7%), anxiety (19.8%), and insomnia (8.3%) among 1,379 Italian HCW during the outbreak.^8^ Incidences of psychological distress were higher among frontline HCW, similar to data published from China.^9^ The main factors associated with stress disorders included supporting the challengingly high number of critically ill patients, scarcity of intensive care beds, unfamiliar treatment strategies, feelings of inadequacy, uncertainty about pandemic duration, the risk of infection and deficiency of personal protective equipment, high-workload shifts, and physical distress related to the need to use heavy protective uniforms.^10^–^12^ Contagion was high among HCW, especially during the first weeks of the outbreak, due to scarcity and non-optimal use of personal protective equipment. To date, more than 25,000 HCW have been infected and 163 doctors have died, many of them working on the frontlines.^13^ Moreover, in light of concerns regarding infection transmission to family members, many health workers decided to stay away from their homes, further complicating the challenges related to the lockdown, such as closed schools and separation from grandparents and other relatives.

While the number of COVID-19 patients was rapidly increasing, Italy saw a drastic decrease in hospital admissions for other pathologies, since patients were afraid of contracting the infection at the hospital. Recent Italian reports document an almost 50% decrease in myocardial infarction admissions for acute coronary syndromes compared to equivalent periods in previous years,^14^ with a subsequent increase in severe cardiac complications and a 3-fold higher cardiovascular mortality rate.^15^

## LACK OF DATA

Facing a novel virus, the primary challenges were related to the lack of data regarding diagnostic criteria, disease management, and treatment options. Diagnostic challenges due to the limited availability of tests, slow processing of samples, and low reliability of some diagnostic kits created anxiety among patients and HCW. To avoid nosocomial spread of the virus, “grey” departments were established for patients with high clinical suspicion; nevertheless, it was almost impossible to prevent in-hospital spread.

Given the limited clinical experience and lack of evidence-based data, new channels for relaying updates emerged; doctors who first started seeing COVID-19 patients primarily shared their experience and findings through Internet pathways. We followed daily updates by attending videoconferences and open webinars to discuss emerging findings, including new clinical signs, typical radiologic patterns, laboratory findings, common complications, and possible treatment strategies. Additionally, open groups on the social networks and dedicated forums were created, reaching up to 100,000 doctors who used these platforms for consultation and exchange of essential real-time information.^16^

As clinical experience advanced, knowledge accumulated about the clinical course of COVID-19 and the essential role played by cytokine storm and coagulopathy for disease progression and complications.^17^–^19^ Given the novelty and the rapid spread of the virus in Italy, most pharmacological treatments were initially based on compassionate and “off-label” use, with wide heterogeneity between centers.^20^ As clinical evidence solidified, protocols became more uniform and included a combination of antimicrobial drugs (antiviral, antibacterial, and antimalarial), anti-inflammatory and immunomodulatory drugs, anticoagulants, and, later, convalescent plasma. A comprehensive management algorithm was recently published by Galluccio et al.,^21^ summarizing the timing indication for each treatment, based on clinical features and laboratory and imaging findings.

## THE IMPORTANCE OF EARLY TREATMENT AND EARLY CONTAINMENT

**Figure 1:**
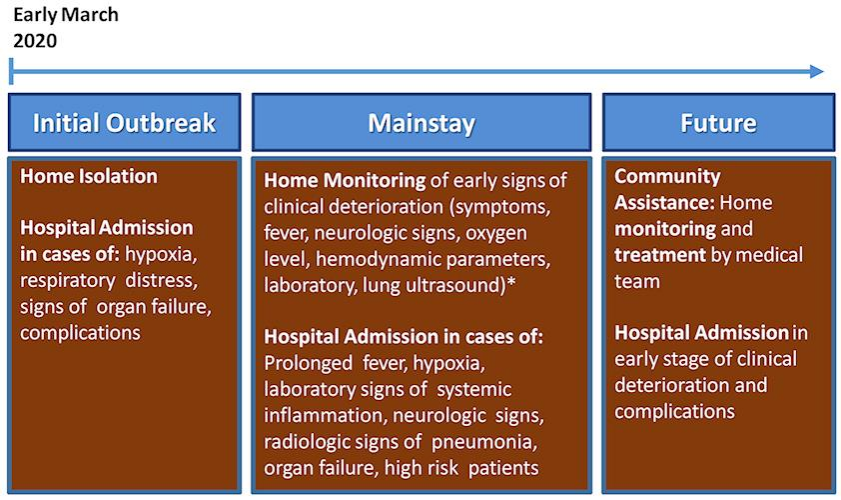
Progression of Management Strategies to COVID-19 Patients in Italy and Future Perspectives * Executed by dedicated medical teams, “USCA—Unità Speciale di Continuità Assistenziale,” and independently organized by the regional local health public body.

Although no targeted treatment is yet available, following the first weeks of outbreak it became clear that recognition of early “warning signs,” and thus early treatment, could drastically reduce disease progression, complications, and mortality. In fact, many critically ill patients developed initial symptoms several days prior to hospital admission and exhibited tardive dyskinesia and sudden clinical deterioration associated with a hyper-inflammatory state.^17^,^21^ Furthermore, many patients presenting with only mild symptoms were found to have a significantly reduced oxygen saturation level and extensive pulmonary damage. In view of the importance of identifying patients in the early stages of disease, an open letter signed by more than 100,000 Italian doctors was sent to the Italian Ministry of Health, asking for a strengthening of community assistance and the telemedicine infrastructure to allow home surveillance and care of COVID-19 patients.^22^ From the end of March 2020, many Italian regions started building dedicated medical teams (called “USCA”—“Unità Speciale di Continuità Assistenziale”), assigned to monitor COVID-19 patients at home through daily calls or home visits. They evaluated clinical parameters (e.g. symptoms, fever, hemodynamics, and neurologic state), and in selected patients laboratory exams and lung ultrasound, which led to hospital admissions earlier in the course of the disease, resulting in a significant decrease in the number of critically ill patients. In view of the concrete risk for a second wave, we believe that there should be more emphasis placed on community assistance, focusing on clinical surveillance and early pre-hospitalization treatment, in order to prevent repeated overcrowding of hospitals and healthcare system collapse. A schematic representation of the COVID-19 management approach—initially, in the midst of the pandemic, and looking ahead—is shown in Figure 1.

## PERSONAL PERSPECTIVE

The COVID-19 outbreak led to an unprecedented health crisis in Italy, as for the rest of the world, with severe consequences on personal and public health, substantial psychological, social, and economic repercussions, and considerable challenges for governments and HCW. Although errors were undoubtedly made at the beginning of the outbreak that significantly contributed to extended illness and the high number of deaths, the Italian government, the national healthcare system, and Italian citizens have reacted with purpose and made important decisions that allowed Italy to gradually recover from the severe crisis.

The decision to impose extensive and national lockdown measures, despite the psychological, social, and economic impact, has permitted significant containment of the virus spread, preventing additional pressure on the healthcare system (which had already exceeded its maximum capacity in many places), and limiting further high morbidity rates and numerous deaths. Moreover, primary importance is attributed to the cooperation of citizens in following the rules, fast reorganization of hospitals in response to the extraordinary workload, broad and real-time sharing of medical findings and updates, strong cooperation between doctors, and the community’s sense of solidarity.

In Italy, there is still much to be done to prevent recurrences, and focus should be on preventing new outbreaks and reinforcing early treatment. Social distancing measures, extensive testing, fast and comprehensive tracing of new cases, and isolation of COVID-19-positive patients in dedicated recovery centers can prevent further widespread diffusion of the virus and the need for more stringent actions. Resources should be invested to strengthen the national healthcare system with an emphasis on community assistance, and digital and telemedicine infrastructures. We believe that it is essential to extend and reinforce outpatient care to prevent clinical deterioration of many patients, reduce hospital admissions, and avoid hospital overcrowding as well as the related consequences on the public healthcare system, non-COVID-19 patients, and HCW.

## CONCLUSION

The COVID-19 outbreak in Italy demonstrated to the world the destructive effects that a highly contagious virus can have on unprepared healthcare and social systems. The high number of patients needing hospital treatment can lead to the overcrowding of hospitals and a very real consequent risk of inadequate treatment of both COVID-19 and non-COVID-19 patients. Containment measures are fundamental to prevent disease dissemination, while monitoring and early treatment are essential to prevent complications and reduce mortality. Italy introduced serious lockdown measures and is now gradually recovering, but the deep wounds incurred by the pandemic have also provided important lessons and insights. We hope that the lessons learned will contribute to the prevention of similar tragedies in the future.

## Abbreviations

COVID-19: Coronavirus disease 2019
HCW: healthcare workers
ICU: intensive care unit.
